# A prospective cohort study of school-going children investigating reproductive and neurobehavioral health effects due to environmental pesticide exposure in the Western Cape, South Africa: study protocol

**DOI:** 10.1186/s12889-018-5783-0

**Published:** 2018-07-11

**Authors:** Shala Chetty-Mhlanga, Wisdom Basera, Samuel Fuhrimann, Nicole Probst-Hensch, Steven Delport, Mufaro Mugari, Jennifer Van Wyk, Martin Röösli, Mohamed Aqiel Dalvie

**Affiliations:** 1 Centre for Environment and Occupational Health Research, School of Public Health and Family Medicine, Faculty of Health Sciences, University of Cape Town, Anzio Road, Cape Town, South Africa; 2 Swiss Tropical Public Health Institute, Basel, Switzerland; 3 University of Basel, Basel, Switzerland; 4 Department of Paediatrics and Child Health, University of Cape Town, Cape Town, South Africa; 5 Hair and Skin Research Laboratory, University of Cape Town and Groote Schuur Hospital, Cape Town, South Africa

**Keywords:** Pesticides, Low exposures, Reproductive health, Neurobehaviour, Co-exposures, School-going children, Rural communities, Endocrine disruption, Air and water

## Abstract

**Background:**

Research on reproductive health effects on children from low-level, long-term exposure to pesticides currently used in the agricultural industry is limited and those on neurobehavioral effects have produced conflicting evidence. We aim at investigating the association between pesticide exposure on the reproductive health and neurobehavior of children in South Africa, by including potential relevant co-exposures from the use of electronic media and maternal alcohol consumption.

**Methods:**

The design entails a prospective cohort study with a follow-up duration of 2 years starting in 2017, including 1000 school going children between the ages of 9 to 16 years old. Children are enrolled with equal distribution in sex and residence on farms and non-farms in three different agricultural areas (mainly apple, table grapes and wheat farming systems) in the Western Cape, South Africa. The neurobehavior primary health outcome of cognitive functioning was measured through the iPad-based CAmbridge Neuropsychological Test Automated Battery (CANTAB) including domains for attention, memory, and processing speed. The reproductive health outcomes include testicular size in boys and breast size in girls assessed in a physical examination, and blood samples to detect hormone levels and anthropometric measurements. Information on pesticide exposure, co-exposures and relevant confounders are obtained through structured questionnaire interviews with the children and their guardians. Environmental occurrence of pesticides will be determined while using a structured interview with farm owners and review of spraying records and collection of passive water and air samples in all three areas. Pesticide metabolites will be analysed in urine and hair samples collected from the study subjects every 4 months starting at baseline.

**Discussion:**

The inclusion of three different agricultural areas will yield a wide range of pesticide exposure situations. The prospective longitudinal design is a further strength of this study to evaluate the reproductive and neurobehavioural effects of different pesticides on children. This research will inform relevant policies and regulatory bodies to improve the health, safety and learning environments for children and families in agricultural settings.

## Background

Chronic health effects resulting from agricultural pesticide exposure, especially at an early stage of development is an important public health concern globally [1, 2]. Neurotoxic effects leading to learning and developmental disabilities as well as male and female reproductive and developmental adverse effects, are of particular concern with respect to exposure to pesticides for example in the chemical group of the organophosphates, carbamates and pyrethroids [3–6]. Several of these pesticides are hormonally active and listed as Endocrine Disrupting Chemicals (EDC’s), which alter biological and developmental functioning at low levels [6, 7]. These harmful pesticides may be absorbed into the organ tissues via ingestion of drinking water, food, inhalation of spray drift or dust and via absorption through skin [7–9].

South Africa, an upper middle-income country has the highest application rates of pesticides in Sub-Saharan Africa with over 3000 different types of pesticide product formulations registered, including the possible neurotoxic and EDC’s active ingredients bifenthrin, chlorpyrifos, cypermethrin and mancozeb [10, 11]. The Western Cape is an important crop farming sector in South Africa. Most of the pesticides including herbicides and insecticides are applied during the summer season [4]. Amongst the number of pesticides detected in the Western Cape area’s surface and ground water that includes drinking water, are chlorpyrifos, deltermethrin, and endosulfan. Endosulfan was most often detected with levels exceeding the World Health Organisation (WHO) standards of 0.1 μ/L for health and safety [12, 13]. High levels of endosulfan metabolites have also been detected in farm workers and residents of the rural Western Cape [13–15].

A cross-sectional study in the rural Western Cape on the reproductive health and development of school boys found different levels of reproductive hormones, lower sexual maturity ratings and anthropometric measurements, in boys who lived on farms compared to those who did not [16]. A case-control study in the Eastern Cape of South Africa found significant associations of birth abnormalities in the offspring of women exposed to agricultural chemicals during pregnancy, including various organophosphates, blue death (a mixture of carbaryl, carbufuran and campechlor/ toxaphene- banned in South Africa since 1970) and other insecticides [17]. Other than the case control study and the cross-sectional studies aforementioned there are no other studies amongst children in South Africa assessing the causal link between pesticide use and health outcomes. Data is especially limited on longitudinal studies amongst low to average exposure to pesticides and its effects on these health outcomes.

To understand the health effects of pesticides requires a better understanding of other factors affecting the physical and neurobehavioral development. Various studies observed associations between electronic media (e-media) use and behavioral patterns including inattention and wellbeing, mostly attributed to use of mobile phones than to radiation exposure [18–21]. Mobile phones are now as common in South Africa as is in America, and mobile phone usage in daily life is common, especially among adolescents. South Africa has 150 mobile phone use subscribers per 100 people, compared to 91 in developing countries, and 71 in Sub-Saharan Africa [22].

Furthermore, South Africa’s Western Cape Province, has the highest rate of Fetal Alcohol Syndrome Disorder (FASD) in the world, with rates higher than 46 cases per 1000 births in recent studies [23]. Previous studies have shown that 46 to 51% of rural woman drink during pregnancy [23]. There are several challenges associated to these high rates particularly relevant in the context of the study areas in the Western Cape: the drinking situation was declared a public health challenge as the fight against alcohol abuse dates back to colonialism in the history of South Africans; no labor laws existed during the apartheid regime, and wages for farm laborers was remunerated in alcohol rather than money, referred to as the Dop (Afrikaans translation for drink) System [24]; FASD is associated 45 times higher amongst woman with lower socio-economic status (SES) than those in middle and upper SES [23]; and the agricultural sector in South Africa is the largest single employment sector, especially for women [25].

The primary aim of this prospective cohort study is to determine the association of agricultural pesticide exposure with reproductive development and neurobehavior of children in the rural Western Cape, South Africa, independent of co-exposures from use of e-media and maternal alcohol consumption. The secondary aim is to investigate associations of these co-exposures on reproductive, neurobehavioral development and well-being of children.

## Methods/design

### Study design

**Fig. 1:**
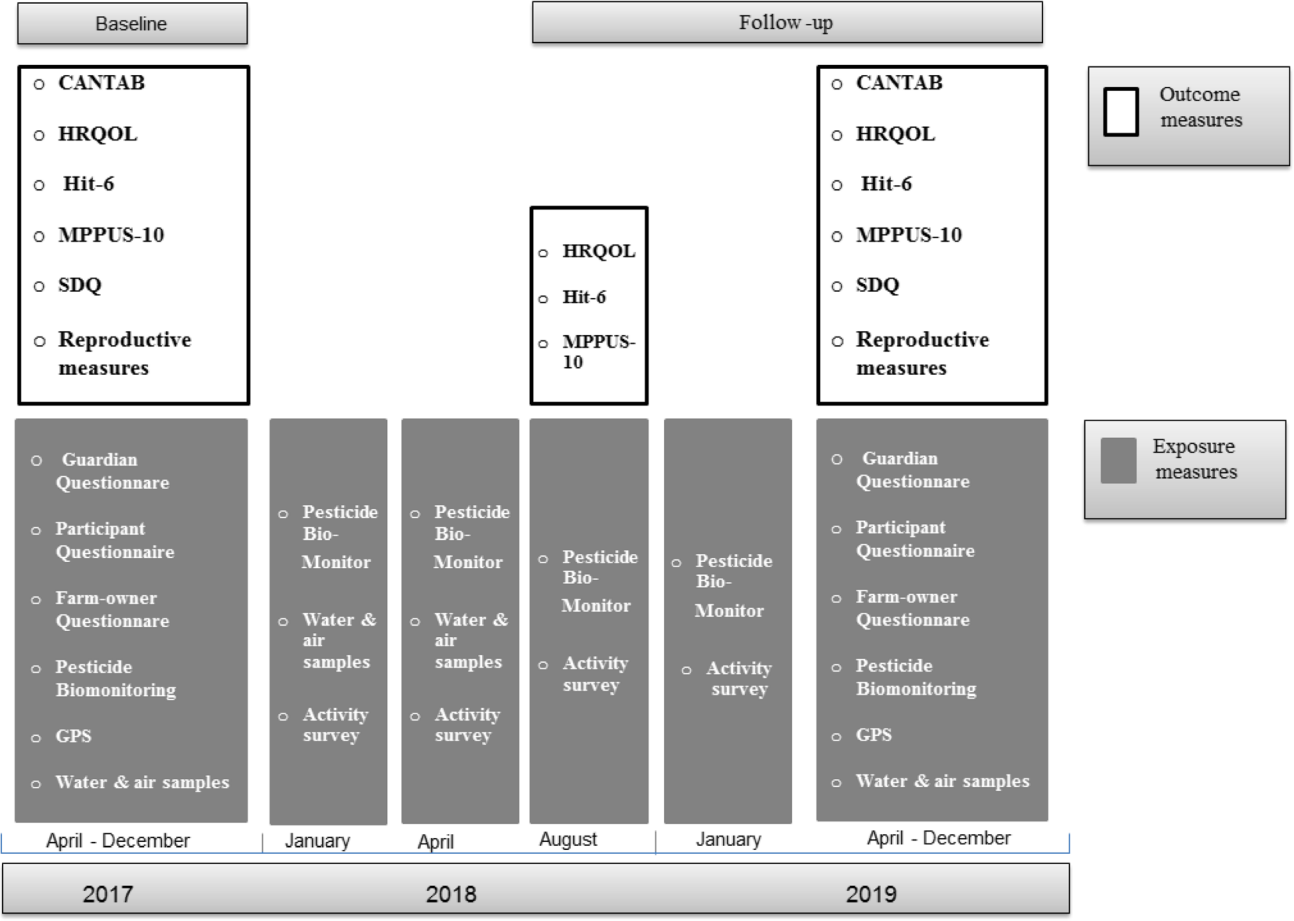
Illustration of the data collection exposure, health outcome tools and timeline in the cohort study

This research study has a longitudinal design comprising a baseline and a follow-up examination of a cohort of 1000 school-going boys and girls from the rural Western Cape of South Africa. The study design is illustrated in Fig. 1 and data collection tools described in detail under the “data collection tools” section below. Study participants are examined at baseline in 2017 and at follow-up in 2019 using the same exposure survey and pesticide biomonitoring exposure measures, as well as the same health outcome measure tools including the CANTAB, reproductive measures including i) Tanner staging - a physical examination of the male and female reproductive system (measuring sexual maturity) ii) reproductive hormone levels and iii) anthopometric measurements, Health Related Quality of Life (HRQOL, also referred to as KIDSCREEN), Headache Impact Test (HIT-6) and Problematic Mobile Phone use (MPPUS-10).

**Fig. 2:**
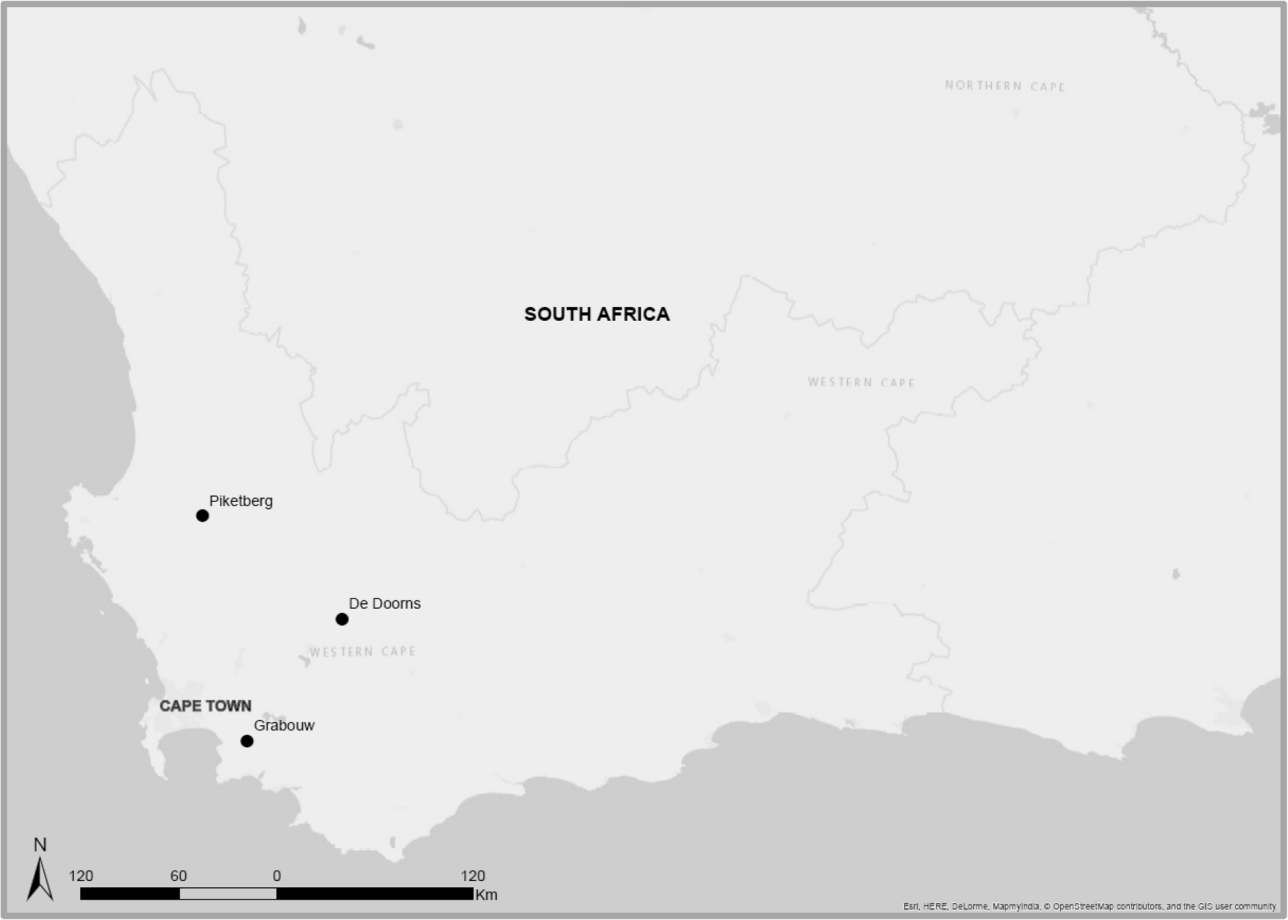
Map of the three study areas, Piketberg, DeDoorns and Grabouw, in the WC of SA Map made by SM using ArcMap 10.5, © OpenStreet Map Contributors and GIS Community

This data collection will take place in a suitable setting during school visits. The participants parent or gaurdian are also visited, to conduct an exposure survey with one health outcome tool about their child, the Strengths and Difficulties Questionnaire (SDQ). Additionally, water and air samples will be collected for 1 year (July 2017–June 2018) and Global Positioning System (GPS) coordinates will be taken both at baseline and at follow-up. The farmers of the surrounding farms to the schools and those farms on which the children live, are contacted to conduct a farm survey on pesticide usage (November 2017–June 2019).

There will be four-monthly follow-ups in 2018 of pesticide exposure measures including biomonitoring (urine samples), and an exposure acitivity survey for the participants only. There will be one follow-up during 2018 for participants on three health outcome tools, the KIDSCREEN, HIT-6 and MPPUS-10.

## Study area

The study is conducted in three agricultural areas: the Hex River Valley (table grapes), Grabouw (apple and stone fruits) and Piketberg (wheat and fruit) Fig. 2. The three study areas were selected according to: (i) its intensive agricultural activities applying large amount of pesticides; (ii) pesticides previously detected in the environment; and (iii) high levels of pesticide metabolites measured in workers and residents from these areas [12].

## Sampling

We recruited 1000 children aged 9 to 16 years from schools in the three study areas. The children were enrolled with equal distribution per area, per gender, as well as an equal number of those living on farms and not living on farms. Of the 32 existing schools in all three study areas, only primary, intermediate and combined schools were contacted to prevent loss to follow-up (i.e., children from high schools would have left school before the follow-up examination in 2019). Of the 22 intermediate schools, 12 were willing to participate and four of the seven combined schools were willing to participate.

The principals and governing bodies of these 16 schools were contacted and informed about the study and seven agreed to participate. In these seven schools, information sheets about the study and the role of the school in this study together with permission letters was then sent to the parents or guardians of all the school children in grades four to nine via their children. The letter served as an invite to participate in the study which had to be indicated by signing and providing detailed contact information. In schools where the number of consenting parents or guardians exceeded the number of children targeted, random systematic sampling was to be used to select the children. Consent from parents or guardians who responded to the study invitation were obtained through home visits.

The grades were expanded from only including 4, 5 and 6 to include grades 2, 3, 7, 8 and 9. Learners from these selected schools were found in these lower and higher grades while still fitting the age criteria, 9–16 years, for the study.

## Data collection tools

### Neurobehavioral health outcomes measurements

#### Cognitive assessment

The online CANTAB Connect Research, developed by Cambridge Cognition, comprise several domains of cognitive testing [26]. The CANTAB is specifically sensitive to changes in neuropscyhological performance and has been applied in over 1750 peer reviewed publications of both human and animal studies [27–29]. Cognitive domains including processing speed, attention, and memory were selected for testing in relation to targeted areas of pesticide and alcohol neurotoxicty. Two tests per domain are selected to measure specific cognitive functions within each of the three domains as shown in Table 1 [30–32]. The CANTAB has different levels for each test to accommodate age variation. The lower levels in each test were selected for this studies age range. Each test is presented to study participants through an iPad (Apple Air, 9.7″) with the installed CANTAB software during the school visit. The time to complete the whole battery requires 40 min from each participant. Each CANTAB test measures cognitive functionining by recording several outcome performance scores on each task including latency and accuracy.

**Table 1:** Cognitive domains and tests performed within the CANTAB cognitive assessment battery

	Cognitive domain	Test	Cognitive function	Outcome	Duration of test
1	Processing speed including visual motor integration	Reaction Time (RTI)	Perception of visual stimuli, response to visual stimuli and execution of motor action	movement time, reaction time and response accuracy;	6 min
		Motor Screening (MOT)	Sensorimotor or perceptual motor speed and comprehension difficulties	Time lapse between display to response; number of correct and incorrect responses	2 min
2	Memory including executive Functioning	Spatial Working Memory (SWM)-	Manipulation of visuo-spatial information, executive demands of strategy (reasoning, decision making and behaviour), parts of short-term memory (holding) concerned with immediate conscious perceptual and linguistic processing	Visits, re-visits and searches for boxes	5 min
		Paired Associate Learning (PAL)	Visual memory and new learning, episodic memory (collection of past, personal experience that occurred at a particular time and place with associated emotions)	Incorrect selection, adjustment, problem solving and memory of selection	8 min
3	Attention	Attention Switching Task (AST)	Attentional set-shifting, cognitive flexibility and lateralization	Congruency and latency during change of instructions	8 min
		Rapid Visual Information Processing (RVP)	Sustained attention and continuous performance, impulse control or inhibition	Sensitivity to target and correct responses	7 min

#### Health related quality of life (HRQOL)

The comprehensive KIDSCREEN tool that measures HRQOL and wellbeing in the areas of physical, psychological, relational support and school enviroment is administered to the study participants [33]. This tool has three different versions and for the purpose of this study’s time constraints, the brief general ten question tool using a five point likert scale for response, requires five minutes to complete.

#### Problematic mobile phone use (MPPUS-10)

The MPPUS-10 is a tool to measure problematic aspects of mobile phone use related to addiction (‘withdrawel’, ‘loss of control’, ‘negative life consequences’ and ‘craving’) and related to social components (peer dependence) [34]. A recent study on cell-phone exposure in adolsecents observed associations of MPPUS-10 with impaired psychological well-being, impaired parent and school relationships and more behavioural problems [18]. Study participants need about 5 minutes to fill in the ten items.

#### Headache impact test (Hit-6)

Headaches have been reported to be associated with cell-phone usage [35–37]. To address this question the HIT-6, a brief and validated tool to assess for the severity of headaches, will be applied. This six question tool, uses a five point likert scale which will take 5 minutes to complete.

#### Strengths and difficulties questionnaire (SDQ)

A brief version of the SDQ is administered to the guardian of the child to screen for any behavioral and affective problems in the child [38]. This widely used tool consists of five scales assessing emotional symptoms, conduct problems, hyperactivity, peer problems and prosocial behaviour on five items each answered on a three point Likert scale.

### Reproductive health outcome measurements

#### Blood reproductive hormones

During the baseline and follow-up examinations, a qualified nurse will collect early morning (before 9 am) whole blood samples (5 ml) from 500 male participants. A sample size of 500 boys (based on findings from previous cross-sectional study [16]) is sufficient to assess differences between farm and non-farm boys. Girl participants were not sampled because of limited funding and the reality that they were less likely to undergo phlebotomy than boys. All the blood samples collected from the study site were transported to the National Health Laboratory Services (NHLS) laboratory based at the Groote Schuur Hospital in Cape Town within 24 h for analysis. Blood samples will be analysed for baseline follicle stimulating hormone (FSH), luteinizing hormone (LH), testosterone, estradiol (E2) and sex-hormone binding globulin (SHBG). Baseline hormones will be compared to age-related laboratory norm using the same assays. LH, FSH, E2 and testosterone are markers of reproductive function in boys as they are part of the Hypothalamic Pituitary Gonadal Axis and their secretion can be altered by hormonally active pesticides [5]. Significant alterations in the levels of these hormones may signal possibilities of estrogenic, anti-androgenic or other in vivo endocrine disrupting effect [6]. SHBG is measured to correct for testosterone. The NHLS laboratory does not currently have the capacity to measure inhibin, the other important male reproductive hormone that suppresses FSH for homeostatic control.

#### Physical examination (reproductive assessment and anthropometry)

Trained and qualified male and female nurses will physically examine the boys and girls respectively in a private room at baseline and follow-up and record the information onto a structured data collection form. Height, weight, secondary sexual characteristics and sexual maturity rating (SMR) will also be recorded [39]. The SMR will be derived by assessing penis development (testicular volume) in boys and breast development (breast size) in girls (129). Testicular volume in boys will be assessed using a standardised set of wooden testicular beads called an orchidometer [40].

Genital anatomical abnormalities including the presence of congenital hydrocoeles, undescended testes, congenital inguinal hernias and hypospadias, and the presence of infection and previous injury will also be assessed for in boys. Additionally, testicular consistency will be recorded for boys. For girls, age at menarche, length and frequency of mensuration and breast development or anatomical abnormalities will also be recorded.

Height and weight will be recorded according to standardised methods and using calibrated instruments.

### Assessment of self-reported exposures and other relevant information

#### Participant questionnaires

The questionnaire administered to participant learners at baseline and follow-up include the following exposure sections:

i. Pesticide Exposure: currently living on a farm or not, recent pesticide contact include seeing and smelling pesticides, swimming in nearby dams or rivers and eating crops from the vine
ii. Farming Activities: involvement with farming activities like picking fruit, spraying, cleaning or burning containers

Confounding variables to the health outcomes in this participant questionnaire include:

iii. Injury and Other Lifestyle Activities: head injuries, sleeping difficulties and substance use.
iv. Electronic Media Use: GERoNiMO: Generalised EMF Research using Novel MethOds
  (including cellphones). To ascertain exposure amongst adolescents in these communities, the European Union project questionnaire, GERONIMO will be administered to enquire about whether they do use electronic media, the type of media usage, activities that they engage with on electronic media devices and the specific time spent engaged with these activities. This tool will be administered before using the MPPUS-10 described under outcome measurements and only those who indicate that they use a mobile phone will complete the MPPUS-10.

The brief pesticide exposure activity questionnaire administered to participants during the four monthly follow-ups include sections (i) and (ii) from the participant questionnaire.

#### Guardian questionnaires

The questionnaire administered to parent or guardian includes the following exposure sections:

i. Pesticide and Household Exposure: previous and current work and residential location, history and current exposure to pesticides during pregnancy, household chemical exposure and childhood pesticide poisoning
ii. Child Residential History: pesticide exposure in both their current and previous residence

Confounding variables to the health outcomes in this participant questionnaire include:

i. Childhood Development: including birth complications and developmental milestones of the child
ii. Medical History: including hospitalisation, diagnoses and medication
iii. Child’s Diet and Nutrition
iv. Maternal Smoking and Alcohol Consumption

#### Farm-owner questionnaire

The farm-owner questionnaire aims to characterise farm activities in three study areas (apple, table grapes and wheat production systems). Therefore, a sub-sample of 20 farms will be selected in each area from the list of farms where children participating in the cohort study are living. Farm owners will be contacted via phone and a meeting will be arranged. If they are willing to participate they will be required to sign an informant consent form to agree to be part of the study to ensure confidentiality of personal data. The data on pesticide use per crop will then be used to develop spatio-temporal crop-exposure matrices (CEMs). The model will provide information on the exposure to individual agricultural pesticides according to the distance to agricultural fields and the season of the year [41].

#### Pesticide biomonitoring

Spot sampling of urine will be conducted per study participant by professional nurses at each sampling time point. There are five sampling time points which are, the baseline in the first year, three times in the second year and during follow-up in the third year of this project to determine short-term pesticide exposure. Approximately 8 ml if urine will be collected from each participant. Each participant’s urine sample will be separated immediately after collection at the collection point into four 2 ml cryovials with color coded caps and stored on dry ice in a cooler box for delivery to the Hair and Skin Laboratory located at Groote Schuur Hospital, University of Cape Town. A cold chain of 2 to 8 °C will be maintained from the moment of urine seperation into cryovials at collection point within 24 h until storage at − 20 °C at the laboratory until they are analysed.

A hair sample, at least 200 mg of hair, will be collected from each participant at each time point in the study, time points stated above, for determining long-term exposure to pesticides. The hair sample will be stored in a aluminium foil at room temperature until they are analysed. This sample will also be analysed by the Hair and Skin laboratory at the University of Cape Town.

The Hair and Skin laboratory will extract and analyse parent pesticides and their metabolites in the urine and hair samples. Currently analysis will be done on commonly used pesticides in the study areas. The initial pesticide analyses will focus on organophosphate metabolites, dialkyl phosphates (DAPs) and pyrethroids (screen) followed by other commonly used pesticides and more specific analyses for individual active ingredients.

In-house validated methods for extraction and analysis of DAPs in urine and hair will be used for each participant. Briefly, DAPs extraction and analysis in urine involves thawing urine samples, one color coded 2 ml vial for each participant in a water bath. A 1 ml volume of the urine sample will be aliquoted into a glass test tube and mixed with a DAP internal standard mixture. The mixture is freeze dried overnight, resuspended in acetonitrile and mixed by vortexing and sonication. The mixture is centrifuged for 10 min at 4000 g and the the supernantant transfered into a glass test tube and dried with a vacuum concentrator. The dried mixture is reconstituted in 200 μl of mobile phase and 10 μl analysed using liquid chromatography-tandem mass spectrometry (LCMS/MS).

DAPs extraction and analysis in hair involved weighing 100 mg of hair into a Omni tube, washing the hair wit 1 ml water followed by pulversing the hair in 1 ml of water using an Omni BeadRuptor. The pulverised mixture is centrifuged and the supernatant is collected, filtered and subjected to LCMS/MS analysis. Methods for analysis of other pesticides and their metaboolites are currently being developed.

#### Assessment of yearly variation of pesticides in air and water

We will assess spatial and seasonal variations of pesticide levels in the atmosphere (over six two-month sampling rounds) and in the aquatic environment (12 one-month sampling rounds) from July 2017 to June 2018. Passive air sampling will be conducted using a total of 36 polyurethane foam disks (PUF-PAS) to sample current used pesticides and 12 PUF-PAS to sample organochlorine pesticides. Samplers will be deployed at two locations in each of the three study areas (one within 50 m to agricultural fields and one > 100 m away in a more urban village environment). Of note, PUF-PAS to measure organochlorine pesticides are only deployed at the location within 50 m to agricultural fields [42]. Given that there are uncertainties regarding the efficiency of sorption to PUFs of polar compounds such as pesticides, three XAD-PAS discs will be additionally deployed at sampling location within 50 m to the farm for 6 months to validate the PUF-PAS sampling systems [42].

Immediately after collection, the PUF-PAS or XAD-PAS samples will be put into a cool box (max 8 °C) and transported to the Chemical Engineering Laboratory at UCT where they will be stored at − 20 °C. In addition, one blank will be distributed in each round for both current used pesticides and organochlorine pesticides. PUF disk samples including field blanks will be transported to the Research Centre for Toxic Compounds in the Environment (RECETOX) in the Czech Republic for analyses. We will target 30+ currently used pesticides (registered and banned in South Africa) in addition to selected organochlorine pesticides which are banned for use in agriculture but may persist in the environment.

Passive water sampling will be conducted at one point downstream of the farming area within the Krom River, Hex River and Berg River located in Grabouw, the Hex River Valley, and Piketberg, respectively. Styrenedivinylbenzene (SDB) disks will be used which allows for continuous time-averaged water sampling at a monthly interval (sampling will be for 2 weeks each month) [43]. After 2 weeks, the SDB disks will be collected and transported to the Chemical Engineering Laboratory at UCT where they will be stored at − 20 °C. In addition, one duplicate will be distributed in each round for quality control. SDB disk samples including laboratory blanks will be eventually transported to the Swiss Federal Institute of Aquatic Science and Technology (EAWAG) in Switzerland. We will target current used pesticides.

To assess water quality, we will measure water temperature, water level, conductivity and pH when samples are deployed and collected every second week. In addition, monitoring data of daily precipitation and water flow for the three river catchment areas will be accessed from the Department of Water and Sanitation of the Republic of South Africa (DWA).

#### Geographical location (GPS)

GPS coordinates of the participating children’s homes will be collected during the home visits when conducting the guardian questionnaire during the baseline study. This data will be used to calculate proximity to agricultural activities (agricultural land use data are obtained via the Cape Farm Mapper which is a product of the Western Cape Department of Agriculture) which will form part of a pesticide exposure index for each participant. The GPS based proximity index will be more accurate than the one used in a previous study which was based on self reported and tape measurement information [44]. The proximity index, spraying intesity index as determined from spraying records, levels of pesticides in the environmental samples and pesticide bio-monitoring will be used to calculate a pesticide environmetal exposure index for each participant.

### Procedure

All personal interviews using a structured questionnaire were installed on mobile devices using Open Data Kit (ODK) application. The GPS coordinates will also be recorded using the ODK application on the mobile phone. To ensure quality of data collection, standard operating procedures (SOPs) are developed and all field workers are trained over a week prior to data collection which will continue at different points throughout the project. The researchers were trained on the CANTAB by the Cambridge Cognition Company product specialists. Two fieldworkers were hired to conduct the CANTAB with specific criteria for the role and were trained on the CANTAB by the research team. The researcher offered continued support to the fieldworkers during the CANTAB data collection, alongside the guidance of the product specialists. Fieldworkers were hired to conduct the interviews with participants, guardians and farm workers and were trained through workshops and role plays on how to conduct the interview using the ODK software with mobile phones and how to record the GPS coordinates. The study nurses to perform the physical examination were trained by an Adolescent/Child health specialist who demonstrated how to perform the anthropometric measurements, use of the orchidometer and assessment of sexual maturation using visual material. The nurses were further trained by the study co-coordinators on their role and tasks within the study as well as on the content of the tools to administer. After sufficient training for the fieldworkers on both the tools and the study itself, two pilot studies were conducted. The first pilot study to test the content and flow of the questionnaires were with 10 participants (five boys and five girls). The second pilot study was conducted on the first 100 participants to test all measurements and its work flow.

Arrangements are made with each school administration for the best logistics including days, time and place to conduct the study. The work flow on days of testing entails five separate data collection stations within appropriate private and quiet venues in each school. At the first data collection station the participants are informed for the second time about the study and given enough detail on the procedure of data collection. Written assent is required at this station. At the second station, learners are examined by a male/female nurse and have their anthropometric measurements taken. After the examination a spot urine sample is collected from boys and processed for transportation to the testing laboratories. Thereafter, they proceed to the third data collection station where learners complete the CANTAB assessment. The fourth station requires completion of the survey with a fieldworker. The fifth station entails a hair and blood sample by a nurse with the final station creating a space for the learner to debrief if needed and receive a treat for their contribution. All exposure and outcome measurements will require an hour and half from each participant and the study aims to reach 25 participants in 1 day.

Following the first phase of data collection from learners at the school, the second phase includes home visits by the fieldworkers. Here the parent/gaurdian will be interviewed which requires an hour to complete the questionaire. At this occasion the fieldworkers take the GPS coordinates of the study participant’s homes and nearest spraying areas. The three follow-up urine samples between baseline and follow-up and the exposure activity questionnaires will then be administered.

The farm-owner questionnaire is administered three times during the study. The first interview was conducted in November 2017 to characterize the farms according to their production system in place and ask participants to provide a copy of their spraying records for 2016/2017 and a copy of their spraying calendars for 2017/2018. Subsequently, the farms are visited and asked to provide a copy of the spraying records in June 2018 and June 2019.

The air and water sampling are conducted during the first phase of the baseline study between July 2017 and June 2018.

### Data analysis

#### Sample size calculations

##### Neurobehavioural outcomes

The sample size for neurobehavioral health outcomes is determined assuming a 0.2 standard deviation from the median neurobehaviour score in the exposed group compared to the control group. This corresponds to observed differences found in studies investigating environmental exposure on neurobehavioral outcomes, using metabolites as the method to assess for pesticide exposure in the Western Cape of South Africa [45–47] in the United States [48] and in Costa Rica [49, 50]. A sample size of 900 was judged to be adequate with a power of 80% and a 5% level of significance.

##### Reproductive hormone outcomes

Sample size calculations for reproductive outcomes were determined using findings from a previous cross-sectional study conducted in the same study areas [16] that showed differences in the same reproductive outcomes as in this study between farm and non-farm residing boys. A two sample-test of equality of means is used (exposed: control ratio = 2:1, i.e. to ensure that more participants are recruited from pesticide exposed areas, power = 80%, confidence level = 95%). The reproductive outcome requiring the highest sample size to show a significant difference in farm versus non-farm boys is for serum testosterone (one of the five hormones to be measured) for which a sample of 498 (i.e. 332 exposed and 166 unexposed) participants are required to ensures sufficient power for the boys in the study. The sample size calculations for boys were considered applicable for girls as all the clinical outcomes were similar and therefore a sample size of 500 girls was targeted to ensure adequate power to test associations between exposures and outcomes.

### Data monitoring

The data monitoring is independent of sponsors. The field coordinator and fieldworkers upload the surveys to the server, while the PhD and post-doctoral students together with the principal investigators monitor the data when uploaded.

### Statistical analysis

Associations between pesticide exposure levels and the obtained health outcome will be conducted by considering relevant confounders. Outcomes of interest include reproduction and pubertal growth (levels of reproductive hormones; sexual maturity rating, height, weight, BMI). The neurobehavioural primary endpoint is cognitive functioning with two secondary endpoints, psychosocial and emotive functioning, health and well-being. Pesticide exposures of interest include bio-monitoring measurements, and the pesticide exposure indices derived from pesticide related risk factors including proximity to the field, contact with the field, involvement in farming activities and contact with the parent or guardian. Co-exposures of interest include e-media use including owning a smart phone and or electronic media device/s, internet use and specific involvement in internet activities such as online games. Another co-exposure of interest includes alcohol consumption during pregnancy characterised by maternal prenatal, perinatal and postnatal alcohol use. Relevant confounders of interest include medical history and current health status, diet, developmental history and indoor chemical use/pollution.

Pesticide exposure will be characterised and compared according to following five different levels: (i) self-reported exposure obtained with the participant questionnaire (e.g., reported behavioral exposure profiles); (ii) self-reported exposure obtained with the guardian questionnaire (e.g., farm worker versus non-farm workers; living on a farm; GPS coordinates of the household and proximity to agricultural fields); (iii) concentration of metabolites and active ingredients measured in urine and hair samples of children; (vi) collected spraying plans and records from farm-owner interviews (to establish pesticide emission profiles for apple, table grapes, wheat and citrus farms and develop a Crop Exposure Matrix (CEM); and (v) measured concentration of active ingredients in passive air and water samples.

Firstly, cross-sectional analyses at baseline and final-follow-up will be conducted. Methods including multiple imputations will be used to address any missing data in the analysis. Further, various types of longitudinal analyses will be conducted. Change analyses will consider whether changes in exposures are related in changes in outcomes. A cohort approach is applied to explore whether exposure at baseline results in new incident cases and provides us the opportunity to assess developmental processes during the time of follow-up. Either clinical case definition is used or in the absence of the criteria for a specific outcome, a priori defined cut-off is used such as the 75th percentile.

Depending on the outcome, logistic, linear or ordinal regression modeling will be conducted.

To maximize power outcomes and exposure, a regression model on a continuous scale (linear/ordinal), will be considered whenever possible. The form of the exposure-response relationships will be explored using polynomial terms or non-parametric approaches (splines). In supplementary analyses, outcomes will be dichotomised and logistic regression modeling will be used. Exposure variables may be dichotomised or categorised for easier communication if suitable.

### Study population

In total, 1400 invite letters were returned from the parents who showed interest to participate. 1001 study participants and their guardian/parents from this pool consented and took part in the baseline examination between April and September 2017. Table 2 gives an overview about the study population and basic demographic information.

**Table 2:** Description of the study population

	Area 1 Grabouw n (%)	Area 2 Piketberg n (%)	Area 3 DeDoorns n (%)	Total n (%)
No. of participants	325(32.5)	303 (30.3)	373 (37.2)	1001(100)
Age categories				
9-11 years	194(59.7)	223(73.6)	175(46.9)	592(59.1)
12-14 years	116(35.7)	79(26.1)	161(43.2)	356(35.6)
15-16 years	15(4.6)	1(0.3)	37(9.9)	53(5.3)
Gender				
Female	170(52.3)	159(52.5)	199(53.4)	528(52.7)
Male	155(47.7)	144(47.5)	174(46.6)	473(47.2)
No. of schools Grade categories	3 (42.8)	2 (28.6)	2 (28.6)	7 (100)
2^nd^-3^rd^	37(11.4)	77(25.4)	49(13.1)	163(16.3)
4^th-^6^th^	235(72.3)	210(69.3)	222(59.5)	667(66.6)
7^th^-9^th^	53(16.3)	16(5.3)	102(27.3)	171(17.1)
Current Farm resident Occupation	202(62.2)	121 (39.9)	142 (38.1)	465(46.4)
Family member works on a farm	199(61.2)	180 (59.4)	281 (75.3)	660(65.9)
Pesticide activities				
Seen pesticide spraying activities in nearby field	278(34.6)	233(76.9)	291(78)	802(80.1)
Helped with cleaning farm equipment	63(85.5)	63(20.8)	97(26)	223(23.2)
Assisted with pesticide storage in the past 7 days	65(20)	49(16.2)	92(24.7)	206(20.5)
Social Media use				
Use a mobile phone	187(57.5)	63(20.8)	68(18.2)	318 (31.7)
Use a smart phone	176(54.2)	48(15.8)	60(16.1)	284(28.4)
Connect to the internet to watch videos	112(34.5)	16(5.9)	17(4.6)	145(14.5)
Connect to the internet to play online games	101(31.1)	6(2)	17(4.6)	124 (12.4)
Connect to the internet to listen to music	111(34.2)	13(4.3)	21(5.6)	145 (14.5)

Table 2 provides descriptive data on the participants in this study, showing they are close to equally distributed amongst the three study areas. The participants age ranges from 9 to 16 years with the highest numbers, almost 60% falling in the younger category of 9-11 years. Their gender is almost equally distributed, with 5% more females than males. The grades range from 2nd to 9th grade, with 66% of the participants falling within the middle category, 4th–6th grade. 46% of the participants live on a farm and 66% have a family member who is a farm worker. Further regarding pesticide exposure, 80% of the participants have said yes to having ever seen pesticide spraying activities in the nearby field; 23% have responded to having helped with cleaning farm equipment in the past; and 20% of the participants have assisted with pesticide storage in the past 7 days. In terms of the participants engagemement with electronic media use, specifically mobile phones, 31% use a phone and of those who use a phone, 89% use smart phones. The majority of these users live in the 1st study area, Grabouw, and are the majority who engage with activities on their phones including watching videos, playing online games and listening to music.

## Discussion

The main strength of this study is its longitudinal prospective design providing the possibility to determine the varying effect of the pesticide exposure, media use and other exposures over different time points. This study also collected detailed objective exposure data on current used agricultural pesticides obtained from urine and hair bio-monitoring, as well as environmental passive air and water sampling in the study areas. This exposure data will enable us to characterise and quantify the level of exposure of the participants and assess their cumulative exposure over the follow-up period.

By combining the biomonitoring with spraying schedules and detailed questionnaire data, a better understanding of critical behaviours for pesticide exposure will be obtained. All study areas are economically important farming areas with intensive use of pesticides, where pesticides have been detected previously and from the results we have attained an equal distribution of participants across these areas. By selecting three different areas where several types of farming products are cultivated, the study offers the possibility to compare the effects of different types and mixtures of pesticides. The study includes almost half of participants from farms compared to children not living on a farm, yielding variations in levels of exposure amongst the cohort. Useful exposure contrasts within the cohort is also demonstrated in other items of the baseline survey, such as seeing spraying activities and engaging with pesticide equipment which was reported by a few participants. About a third of the participants do engage with media use in all three areas, even though these are low income rural areas. Thus, the cohort is well suited to study the effects of uptake of media in adolescence.

The study population has an appropriate age range that includes children in various stages of development which will enable the researchers to investigate the changes in periods of pubertal and neurobehavioral development amongst the distinct groups of exposed children over the two-year period.

Development stage is assessed using standardised methods and complemented with a wide range of hormonal measurements indicative of reproductive development obtained from blood samples. Use of a validated computerised tool to measure neurobehaviour is a further asset of this study. This is the first iPad based study on this topic in a rural setting which will yield evidence of standardizing quality data and reliability for future studies on a large scale. Additionally, the questionnaires are comprehensive for collecting data on children’s diet, socio-economic status, prenatal exposures and family environment to determine any influencing factors on behavior and development. The generation of exposure indices and the area of residence will help in understanding the patterns of lifetime exposure in relation to different environmental factors for e.g. proximity to spraying area.

Furthermore, by including co-exposures, pesticide effects can be studied independent of e-media and alcohol consumption, while synergistic effects can be studied.

Lastly this study carries power for attaining its objectives and methods with the use of a 1000 sample population.

Results from this study will be used to educate the community and government sectors involved in pesticide use and regulation. Suggestions that arise from this study will provide farming communities with awareness of health promotion and prevention strategies. In conclusion the findings from this study can contribute to the improvement and protection of children’s health and development locally and internationally as these pesticides are used globally. This will be the first longitudinal study investigating the reproductive health effects on children of agricultural pesticides in current use and will seek to address conflicting results from studies investigating neurobehavioural effects.

## Abbreviations

ADHD: Attention Deficit Hyperactivity Disorder
ASD: Autism Spectrum Disorder
CANTAB: CAmbridge Neuropsychological Test Automanted Battery
CEM: Crop-exposure matrices
DAFF: Department of Agriculture, Forestry and Fisheries
EDC: Endocrine Disrupting Chemical
FASD: Fetal Alcohol Syndrome Disorder
HIT-6: Headache Impact Test-6
ODK: Open Data Kit
PDD: Pervasive Developmental Disorders
PUF-PAS: Polyurethane foam disks
SDB: Styrenedivinylbenzene
WHO: World Health Organisation
XAD-PAS: XAD resin
